# Craniofacial and three-dimensional palatal analysis in cleft lip and palate patients treated in Spain

**DOI:** 10.1038/s41598-022-23584-0

**Published:** 2022-11-06

**Authors:** María José Viñas, Francesca Galiotto-Barba, María Gabriela Cortez-Lede, María Ángeles Rodríguez-González, Ignacio Moral, Elena Delso, Beatriz González-Meli, Fernando Lobo, José Luis López-Cedrún, David Neagu, Joaquín Garatea, Amaia Garatea, Beatriz Berenguer, Concepción Lorca-García, María Dolores Delgado, Eunate Martí, José Manuel Gutiérrez, Carlos Hernández, Jorge Murillo-González, Concepción Martínez-Álvarez, Elena Martínez-Sanz

**Affiliations:** 1 Facultad de Odontología, Universidad Complutense de Madrid, 28040 Madrid, Spain; 2 Hospital Clínico Universitario Virgen de la Arrixaca, Servicio Murciano de Salud, 30120 Murcia, Spain; 3 Hospital Universitario Miguel Servet, Servicio Aragonés de Salud, 50009 Zaragoza, Spain; 4 Hospital Universitario Infantil Niño Jesús, Servicio Madrileño de Salud, 28009 Madrid, Spain; 5 Complejo Hospitalario Universitario A Coruña, Servizo Galego de Saúde, 15006 A Coruña, Spain; 6 Complejo Hospitalario de Navarra, Servicio Navarro de Salud, 31008 Pamplona, Spain; 7 Hospital General Universitario Gregorio Marañón, Servicio Madrileño de Salud, 28009 Madrid, Spain; 8 Hospital Universitario 12 de Octubre, Servicio Madrileño de Salud, 28041 Madrid, Spain; 9 Hospital Universitario de Burgos, Sanidad de Castilla y León, 09006 Burgos, Spain; 10 Facultad de Medicina, Universidad Complutense de Madrid, 28040 Madrid, Spain

**Keywords:** Anatomy, Diseases, Health care, Medical research

## Abstract

Growth alterations have been described in patients operated on for oral clefts. The purpose of this work was to analyze the craniofacial and palate morphology and dimensions of young adults operated on for oral clefts in early childhood in Spain. Eighty-three patients from eight different hospitals were divided into four groups based on their type of cleft: cleft lip (CL, n = 6), unilateral cleft lip and palate (UCLP, n = 37), bilateral cleft lip and palate (BCLP, n = 16), and cleft palate only (CPO, n = 24). A control group was formed of 71 individuals. Three-dimensional (3D) digital models were obtained from all groups with an intraoral scanner, together with cephalometries and frontal, lateral, and submental facial photographs. Measurements were obtained and analyzed statistically. Our results showed craniofacial alterations in the BCLP, UCLP, and CPO groups with an influence on the palate, maxilla, and mandible and a direct impact on facial appearance. This effect was more severe in the BCLP group. Measurements in the CL group were similar to those in the control group. Cleft characteristics and cleft type seem to be the main determining factors of long-term craniofacial growth alterations in these patients. Prospective research is needed to clearly delineate the effects of different treatments on the craniofacial appearance of adult cleft patients.

## Introduction

Cleft lip and palate is the most prevalent major craniofacial malformation, arising in approximately 1.7 per 1000 live births^1^. It appears when the anlages of the upper lip and/or palate do not fuse properly, and the nasal and oral cavities communicate at birth. The main types of oral clefts are cleft lip (CL), cleft palate (CP, including alveolar cleft, cleft of the secondary palate, and cleft palate only CPO), and combined cleft lip and palate (CLP), which can be either unilateral (UCLP), when only one side is affected, or bilateral (BCLP), if the cleft involves both sides. Clefts can also be complete, when all the structures of the region are cleft, or incomplete, if some of the structures fuse prenatally. In Spain as a whole, prevalence data show a statistically significant decreasing linear trend from 1980 to 2019 for both CL and CP (6.49–3.80 per 10,000), as well as CPO (5.20–3.58 per 10,000), considering the latest recorded information available^2^.

Patients with oral clefts undergo a complex interdisciplinary treatment that includes preoperative procedures and multiple surgical interventions^3^. Among the preoperative procedures, nasoalveolar molding (NAM)^4^ is mainly used in wide clefts to better position the maxillary segments and nose cartilage for surgical repair. In bilateral cleft lip, the columella may also be lengthened. Surgeons perform primary rhinoplasty together with lip repair (including a variety of techniques, ranging from McComb suspension to complete open tip plasty with septal repositioning). Secondary intermediate and definitive rhinoplasty is most often used for severe clefts. Different types of cheiloplasties are used to repair cleft lip, mainly rotation advancement repair (Millard and variations), straight line closure (Fisher and variations), and triangular flap repair (Tennison). For cleft palate repair, surgeons may select several techniques. The most commonly used surgical approach to repair the hard palate includes the elevation of two mucoperiosteal flaps, which are sutured together in the midline (Veau-Wardill-Kilner, Bardach). Other approaches to repair cleft palate include lateral relaxing incisions with no or minimal subperiosteal dissection (Von Langenbeck), sometimes together with intravelar veloplasty (Sommerlad). Surgeons may perform early gingivoperiosteoplasty for alveolar repair to reduce the number of surgeries. However, secondary alveolar bone grafting with cancellous bone obtained mostly from the iliac crest remains the most common procedure. Despite protocolized expert treatment, complications and sequalae are frequent and require specific additional treatment. Symptomatic palatal fistulas are repaired with local or regional flaps depending on size and location. Velopharyngeal incompetence requires coordinated speech therapy and possible additional surgeries adapted to the size, shape, and dynamics of the gap (palatal lengthening [Furlow double opposing z-plasty], pharyngoplasty or pharyngeal flap). If necessary, secondary aesthetic corrections of the lip and/or nose are often performed at the same time as the protocolized procedures in the treatment calendar to further reduce the treatment burden. In general, protocols recommended in Spain to repair oral clefts start at the age of 3–6 months, with primary cheilo-rhinoplasty. Closure of the hard and soft palate is usually performed simultaneously at any time between 6 and 15 months. Secondary surgery for palatal fistulas or velopharyngeal insufficiency is typically performed between 3 and 6 years of age. The alveolar clefts, either primary or residual, are usually treated around the age of 10 years by means of a bone graft, usually obtained from the patient’s hip^5^*.*

It has been claimed that the alterations of mid-facial growth observed in operated cleft patients, which include alveolar arch collapse, midface retrusion, and malocclusion, are caused by previous surgeries^6–9^. These defects are evident in late adolescence, when craniofacial growth is complete, and usually require orthodontic and/or orthognathic treatment. Alternatives to classical surgical procedures aiming to minimize the impact of conventional CP repair on craniofacial growth have been explored in humans^10^, in vitro^11,12^, or in experimental models^13^, but even though the results of these studies are promising, conventional palatoplasty is still the treatment used to close the palate defect.

The craniofacial alterations observed in these operated patients include a diminished mandibular size with a clockwise rotation of the mandible and a characteristic facial morphology with a longer vertical dimension^14^. These patients also present a constricted upper arch and a narrow palatal vault^15^. Several of these features are the result of measuring dental casts taken from the oral cavity of the patients. However, taking dental impressions may imply risks. The use of an intraoral scanner has been shown to be more comfortable in operated UCLP and for patients with repaired CL, in which conventional impressions may be uncomfortable due to scarring of the upper lip^16^. In addition, the use of three-dimensional (3D) virtual models allows palate surface area and volume measurements to be obtained^17^, and the reliability of scoring dental arch relationships using intraoral 3D scans has proved to be superior to indirect digital and plaster models^16^.

In Spain, there is no information on the craniofacial outcomes of adult patients operated on for their cleft in childhood. The present study aimed to analyze the craniofacial and palate morphology and dimensions of a population of young adults whose cleft was repaired in eight different Spanish hospitals, in an attempt to determine the effects of the treatments provided on their long-term craniofacial growth pattern. We also strived to highlight possible differences between different types of clefts. This knowledge may provide insight into the impact of surgery on the long-term craniofacial clinical outcomes of oral cleft patients.

## Results

## Digital models

Table 1 shows the mean and standard deviation of the measurements obtained from the 3D digital model variables in the groups and statistical differences between them. The CL group was the only group showing no significant differences in all the measurements analyzed in the 3D models with respect to the control group. The UCLP and BCLP groups showed a significant decrease in the palatal volume in comparison with the control, whilst the CPO patients had a significant reduction of both the palatal volume and the intermolar width. No significant differences were found between any of the study groups and the control group for the intercanine width and arch depth.

**Table 1:** Comparison of three-dimensional digital model measurements between groups.

	CONTROL (n = 71)			CL (n = 6)			UCLP (n = 37)			BCLP (n = 16)			CPO (n = 24)			(Sig.)
	Mean		S.D	Mean		S.D	Mean		S.D	Mean		S.D	Mean		S.D	
Palatal volume	7371.35	±	1631.65	5776.67	±	1667.11	5762.68	±	1963.54	4575.5	±	1112.67	5990.86	±	1654.51	***^a,b^, *^c^
Intercanine distance	23.74	±	2.02	24.25	±	1.89	23.18	±	2.29	22.62	±	1.81	24.26	±	2.43	NS
Intermolar distance	35.12	±	2.4	32.34	±	2.9	33.17	±	3.8	31.46	±	2.07	31.97	±	4.9	**^c^
Arch depth	23.45	±	1.82	25.04	±	2.98	21.99	±	2.92	21.98	±	2.36	21.99	±	3.82	NS

*Sig.* Statistical significance, *NS* not significant.

*p ≤ 0.05; **p ≤ 0.01; ***p ≤ 0.001;

^a^Controls versus UCLP.

^b^Controls versus BCLP.

^c^controls versus CPO.

## Cephalometric analysis

Table 2 shows a comparison of the skeletal and dental cephalometric measurements between groups.

**Table 2:** Comparison of cephalometric measurements between groups.

	CONTROL (n = 71)	CL (n = 6)	UCLP (n = 37)	BCLP (n = 16)	CPO (n = 24)	(Sig.)
**Maxilla**						
SNA	83.59 ± 3.90	77.5 ± 2.95	75.24 ± 4.56	76.19 ± 4.05	78.38 ± 5.22	***^b,c,d^
Maxillary depth	91.73 ± 2.66	88.17 ± 2.04	86.75 ± 3.94	86.4 ± 4.23	87.39 ± 6.14	***^b,c^,*^d^
Midfacial length	88.96 ± 6.62	80.76 ± 7.22	81.25 ± 8.7	82.68 ± 6.66	84.39 ± 9.33	***^b^,*^c,d^
A-Nasion vertical	1.73 ± 2.65	− 2.06 ± 1.93	− 3.72 ± 4.4	− 4.39 ± 4.6	− 2.45 ± 6.38	***^b,c^,*^d^
**Mandible**						
SNB	81.31 ± 3.39	75 ± 4.05	74.49 ± 5.11	74.75 ± 3.95	75.88 ± 4.79	*^a^,***^b,c,d^
Facial depth	90.63 ± 2.87	86.67 ± 4.03	87.16 ± 4.08	86.56 ± 3.95	86 ± 5.48	***^b,d^,**^c^
Pogonion-Vertical Na	0.94 ± 5.5	− 6.5 ± 7.74	− 5.6 ± 8.2	− 7.3 ± 8.29	− 8.4 ± 11.5	**^b,c^,***^d^
Ramus height	52.9 ± 5.38	46.28 ± 4.73	48.98 ± 6.99	47.01 ± 4.7	49.33 ± 5.56	*^b^,**^c^
Mandibular body length	71.77 ± 8.05	67.25 ± 5.28	66.95 ± 8.61	65.95 ± 8.15	67.2 ± 9.2	*^b^
Effective mandibular length	117.56 ± 8.88	108.28 ± 7.96	114.66 ± 12.44	113.57 ± 10.8	115.75 ± 11.78	NS
**Maxillomandibular relationship**						
ANB	2.23 ± 1.9	2.5 ± 2.5	0.65 ± 3.29	1.44 ± 3.16	2.5 ± 3.18	*^b^
Maxillomandibular differential	28.62 ± 4.29	27.5 ± 5.18	33.41 ± 6.55	30.92 ± 6.55	31.37 ± 6.21	***^b^
**Growth pattern**						
Facial axis	90.27 ± 4.72	84.67 ± 4.89	82.86 ± 5.55	83.27 ± 4.37	82 ± 6.36	***^b,c,d^
Mandibular plane angle	19.57 ± 4.6	24.83 ± 5.19	27.25 ± 6.9	28.8 ± 5.97	30.17 ± 8.88	***^b,c,d^
Gonial angle	121.66 ± 7.5	120 ± 9.78	126.97 ± 7.59	131.25 ± 7.46	129.33 ± 9.95	*^b^, ***^c^, **^d^
**Cranial base**						
Saddle angle	122.56 ± 4.85	123.17 ± 7.05	124.86 ± 6.16	125.00 ± 6.16	124.67 ± 5.83	NS
Articular angle	143.69 ± 7.05	151.67 ± 12.03	146.11 ± 8.14	142.38 ± 6.2	144.83 ± 9.18	NS
Anterior cranial base	70.72 ± 5.55	68.15 ± 3.09	68.43 ± 5.66	68.73 ± 6.4	68.09 ± 5.01	NS
Posterior cranial base	34.51 ± 3.6	31.65 ± 1.24	33.86 ± 4.72	33.76 ± 3.88	36.1 ± 5.59	NS
**Inclination relationship**						
Lower incisor inclination (IMPA)	94.02 ± 6.72	95.83 ± 7.98	85.92 ± 7.62	87.75 ± 7.14	86.79 ± 11.85	***^b^, *^c,d^
Upper incisor/Frankfort plane	111.7 ± 6.22	114.83 ± 9.43	109.3 ± 7.88	105.25 ± 7.15	109.17 ± 5.82	*^c^

*Sig.* statistical significance, *NS* not significant.

*p ≤ 0.05; **p ≤ 0.01; ***p ≤ 0.001.

^a^Controls versus CL.

^b^Controls versus UCLP.

^c^Controls versus BCLP.

^d^Controls versus CPO.

### Maxilla

Regarding the maxillary skeletal variables, the CL group was the only study group that did not differ from the control group. With respect to the control group, the UCLP, BCLP, and CPO groups showed a statistically significant reduction of SNA, maxillary depth, effective midfacial length measurements, and the distance of point A to a vertical line from the nasion.

### Mandible

Regarding mandibular measurements, the CL group only showed a significantly decreased SNB angle. In the UCLP and BCLP groups, the SNB, facial depth, ramus height, and pogonion to vertical nasion measurements were significantly diminished. The mandibular body length was diminished only in the UCLP group with respect to the control. In the CPO group, the SNB, facial depth, and pogonion to the vertical from nasion measurements were significantly diminished with respect to the control group. No significant differences were found between cleft groups in the effective mandibular body length.

### Maxillomandibular relationship

With respect to the maxillomandibular relationship, significant differences were only found in the UCLP group compared to the control group, with a reduction of ANB and an increase in the maxillomandibular differential.

### Growth pattern

While no differences were found in the CL group, the rest of the groups (UCLP, BCLP, and CPO) showed a diminished facial axis and increased gonial and mandibular plane angles.

### Cranial base

No structural differences were found between groups regarding the cranial base.

### Incisor inclination

No differences were found in the CL group with respect to incisor inclination. Lower incisor inclination to the mandibular plane was decreased in the UCLP, BCLP, and CPO groups compared to the control group, while the upper incisor inclination to Frankfort’s horizontal plane was decreased only in the BCLP group.

## Soft-tissue analysis

Table 3 shows frontal, lateral, and submental soft-tissue measurements and comparisons between groups.

**Table 3:** Comparison of soft-tissue measurements between groups.

	CONTROL (n = 71)	CL (n = 6)	UCLP (n = 37)	BCLP (n = 16)	CPO (n = 24)	(Sig.)
**Frontal facial analysis**						
Facial height	131.56 ± 9.84	137.75 ± 9.5	139.92 ± 11.68	138.61 ± 9.18	141.05 ± 14.69	*^b,d^
Facial width	135.56 ± 8.07	144.41 ± 5.3	139.31 ± 9.13	133.54 ± 5.37	139.28 ± 10.93	NS
Facial index	97.01 ± 6.28	96.66 ± 5.16	100.27 ± 7.25	103.50 ± 7.01	101.73 ± 7.16	*^c^
Vertical dimension	186.81 ± 13.44	198.26 ± 15.13	196.02 ± 12.47	194.52 ± 11.05	197.47 ± 15.18	**^b^,*^d^
Width of the mouth	48.81 ± 3.35	50.58 ± 4.13	49.38 ± 4.43	47.12 ± 1.15	48.83 ± 3.69	NS
Intercanthal width	29.54 ± 2.12	31.93 ± 2.90	30.07 ± 2.50	29.04 ± 1.53	29.64 ± 2.90	NS
**Lateral facial analysis**						
Nasal length	49.35 ± 4.27	52.58 ± 4.56	50.92 ± 4.2	51.75 ± 3.36	52.21 ± 4.91	NS
Nasolabial angle	103.84 ± 12.38	99.17 ± 15.81	97.38 ± 18.41	103.5 ± 12.38	109.42 ± 20.65	*^f^
Upper lip position	− 3.40 ± 4.95	− 2.23 ± 2.6	− 2.2 ± 4.03	− 1.4 ± 3.9	− 2.4 ± 4.58	NS
Upper lip length	21.32 ± 2.44	17.65 ± 3.07	19.57 ± 2.99	18.12 ± 2.27	20.83 ± 2.31	*^a,g^, ***^c^
Lower lip length	46.29 ± 4.92	44.35 ± 6.37	49.25 ± 5.98	47.79 ± 6.36	49.44 ± 5.1	NS
**Submental facial analysis**						
Alar base width	35.24 ± 3.85	37.01 ± 5.5	36.39 ± 4.89	39.23 ± 3.65	34.82 ± 2.98	**^c,g^
Nasal height	23.47 ± 2.15	25.87 ± 4.6	23.85 ± 3.39	24.3 ± 2.91	23.24 ± 2.58	NS
Columella height	11.89 ± 1.45	11.75 ± 1.55	11.01 ± 2.62	10.87 ± 2.1	11.54 ± 2.47	NS
Ratio columella height/nasal height	0.51 ± 0.05	0.46 ± 0.04	0.46 ± 0.08	0.45 ± 0.06	0.50 ± 0.1	*^b,c^
Ratio columella height/alar base width	0.34 ± 0.05	0.32 ± 0.03	0.3 ± 0.07	0.28 ± 0.05	0.34 ± 0.08	*^b,c^, *^g^
Right nostril angle	58.73 ± 6.34	59.33 ± 9.61	61.43 ± 13.1	53.13 ± 9.83	58.54 ± 12.48	NS
Left nostril angle	59.12 ± 6.7	57.00 ± 7.69	61.2 ± 11.58	55.47 ± 11.33	61.83 ± 9.43	*^g^
Right nostril area	49.63 ± 11.67	57.44 ± 16.55	52.76 ± 18.32	64.19 ± 18.61	50.44 ± 20.67	NS
Left nostril area	48.87 ± 10.56	48.87 ± 10.56	54.83 ± 20.62	62.28 ± 22.39	50.85 ± 20.77	NS
Nasal deviation	0.55 ± 1.36	3.67 ± 3.14	6.77 ± 3.3	3.2 ± 2.81	2.54 ± 3.8	NS
Nostril angle (R-L differences)	− 0.38 ± 3.72	2.33 ± 9.13	0.22 ± 13.83	− 2.33 ± 9.99	− 3.92 ± 6.07	*^d^
Nostril area (R-L differences)	0.76 ± 5.33	− 1.64 ± 8.74	− 2.07 ± 16.45	1.91 ± 15.76	− 0.41 ± 7.48	NS

*Sig.* Statistical significance, *NS* not significant.

*p ≤ 0.05; **p ≤ 0.01; ***p ≤ 0.001.

^a^Controls versus CL.

^b^Controls versus UCLP.

^c^Controls versus BCLP.

^d^Controls versus CPO.

^e^CL versusUCLP.

^f^UCLP versus CPO.

^g^BCLP versus CPO.

### Frontal facial analysis

In the frontal facial analysis, no differences were found between the CL group and any other group. In both the UCLP and CPO groups, the results showed a significant increase in the facial height and vertical dimension with respect to the control group. The BCLP group showed a significant increase in the facial index. There were no significant between-group differences in the width of the mouth and the intercanthal width.

### Lateral facial analysis

In the nasal lateral analysis, the UCLP group showed a significantly diminished nasolabial angle compared to the CPO group, with no other significant differences. No differences were found between any of the groups in nasal height. Regarding lip analysis from the profile view, the CL and BCLP groups showed a significantly diminished upper lip length compared to the control group, with the CL group showing the greatest decrease. In the BCLP group, this length was also significantly diminished compared to that in the CPO group. There were no significant differences among groups regarding the lower lip length or upper lip position.

### Submental facial analysis

A statistically significantly greater nasal alar base width was found in BCLP patients compared to controls or CPO patients. Both columella height/nasal height and columella height/alar base width ratios were significantly lower in UCLP and BCLP patients compared to the control group. The ratio columella height/alar base width was also significantly lower when BCLP patients were compared with CPO patients. The angulation of the long axis of the left nostril was lower when the BCLP patients were compared with CPO patients. Only the CPO patients showed statistical differences when the right and left nostril angles were compared with the control group. No statistically significant differences were found among groups for other nasal variables (Table 3).

## Influence of the age at surgery on craniofacial growth and facial analysis

The analysis of the relationship between the age in months when cheiloplasty and palatoplasty were performed and the craniofacial growth and facial variables studied showed no significant (*p* = 0.335 and *p* = 0.897, respectively) differences.

## Discussion

The present study describes the long-term craniofacial pattern of previously treated oral cleft patients in Spain when they reached adulthood, including a comparison between four oral cleft types. This means that we are analyzing the consequences of a multidisciplinary treatment that started at least 18 years ago. This reduced the possibility of accessing these patients, which clearly limited the size and gender composition of the sample, especially that of the CL group, and made it impossible to categorize it by sex, which is frequent in the majority of studies^14,18^. The number and gender of the participants in the control group was also limited by the inclusion criteria requirement of having a previous cephalogram for orthodontic diagnosis, which aimed not to subject these individuals to additional radiation, together with the absence of previous orthodontic treatment.

In addition to a study of their cephalometries and facial analysis, this is the first study in the Spanish population of oral cleft patients to use 3D analysis of models obtained through an intraoral scanner. Three-dimensional digital models are useful for diagnosis and the analysis of treatment results^19,20^, and 3D measurements on digital models allow for a better understanding of the shape and size of the palate in cleft patients. Previous studies analyzing palatal volume in 3D models from cleft subjects acquired records indirectly by digitizing plaster casts obtained from conventional impressions. In our study, 3D models were obtained directly from patients by using an intraoral scanner. We also wish emphasize the relevance of including a control group in this study, since there is hardly any information on palatal 3D measurements in non-cleft control groups. The measurements obtained here may serve as a guide for control groups in future research. To analyze the facial soft tissue, we used 2D measurements. This method has limitations versus 3D photogrammetry, which may provide useful information regarding facial analysis, as it gives area and volume measurements^21,22^. Future research should include the use of 3D facial analysis and questionnaires from the patient’s point of view regarding the aesthetic outcome of treatment.

As observed in other studies performed on different cleft types^17,23–25^, our results showed a diminished palatal volume in the UCLP, BCLP, and CPO groups compared to the control group, even when orthodontic expansion of the palate was applied to all these patients during growth. The BCLP group showed a greater alteration of the palate volume than the other groups, since a greater decrease in this measurement was found. A diminished palatal volume implies a reduced space for the tongue, which has functional implications for speech and breathing, among others^24^.

As previously reported^25^, a low tongue posture during growth, due to the decreased space of the palate, may also have favored the development of malocclusion with vertical development, open bite, and posterior rotation of the mandible observed in our patients. In our study, the intercanine width was diminished in all cleft groups, but these differences were not statistically significant. This may be attributable to successful orthodontic treatment, since all the patients of our study were treated orthodontically. The intermolar width was significantly diminished only in CPO patients, which is in concordance with the results obtained by Schultes et al.^26^, who found that adult patients with operated CPO had a smaller transversal space. This has also been observed long after surgery in animal models of CPO^27^.

A hypoplasia of the maxilla has been described previously in cleft patients^3,18,26,28^. In our study, a restriction of the maxillary growth was observed in UCLP, BCLP, and CPO patients, as the SNA measure points out. No differences were detected among the cleft groups in the size or position of the maxilla. There is controversy as to whether this hypoplasia is inherent in the condition of the cleft itself^14,29,30^ or whether it is a consequence of the palatoplasty patients undergo at an early age^14,31,32^. Studies in animal models support the idea that the cleft itself is a cause of the maxillary growth impairment found in cleft patients^27^. Regardless of the etiology of this maxillary development deficit, our study revealed that operated patients with BCLP, UCLP, and CPO reached adulthood with maxillary hypoplasia despite orthodontic treatment and that, interestingly, patients with only CL, even when a lip scar was inevitably present, did not present maxillary hypoplasia.

Paradoxically, although we found a facial growth deficit of the middle third of the face in the UCLP, BCLP, and CPO groups, which could affect the nasolabial angle, the lateral facial analysis of these groups did not reveal significant differences in this measure with respect to the control group. Similarly, the nasal length of these patients was not affected. When comparing the nasolabial angle among cleft groups, a significant decrease in this angle was observed in the UCLP group in relation to the CPO group, which showed the highest mean value in this variable. This might be due to the unaltered lip in CPO patients. The nasolabial angle plays an important role in the analysis of facial harmony in the sagittal plane and is a remarkable measure of the treatment outcome. In our study, the nasolabial angle presented a large standard deviation in all groups, thus indicating that an ideal aesthetic result was not achieved after treatment in many cases. A large deviation from the mean in the nasolabial angle is a common finding in many studies, and, as Bearn et al.^33^ pointed out, although the nasolabial angle is a good measure of the treatment outcome, it does not lend itself to statistical analysis.

A small mandible and a small ramus height have been described in both operated^32^ and non-operated cleft patients^30^. In our study, the distance from the pogonion point to the vertical from the nasion point and facial depth, which reflect the sagittal position of the mandible, were significantly decreased in the UCLP, BCLP, and CPO groups. The more retruded mandible, together with maxillary hypoplasia, would explain the more retrognathic concave profile observed in our patients, which is characteristic of cleft palate patients^3^. In the UCLP and BCLP groups, the mandible had a retruded position and a decreased height of the mandibular ramus. The CPO group differed from the UCLP and BCLP groups in that the size of the ramus height was not affected.

Although the mandibular body length decreased in all groups compared to the control group, this difference was only statistically significant in the UCLP group. On the other hand, the effective mandibular length did not present significant differences in any of the groups, and this may be attributed to the increase in the gonial angle in UCLP, BCLP, and CPO groups. Unlike the rest of the groups, the variables that refer to the mandible showed normality in the CL group compared to the control group, except for the SNB angle, which decreased in this group. Thus, the SNB angle is the only measure that was affected in the four types of cleft. Summarizing, regarding the mandibular characteristics, the groups with the greatest mandibular alteration were the BCLP and the UCLP groups, followed by the CPO group, while the CL group was barely affected. In concordance with our results, minimal skeletal and dental effects have been described in CL patients^34^.

Regarding the maxillomandibular relationship, only the UCLP group showed significant differences when compared to the control group, with a reduction in ANB and an increase in the maxillomandibular difference, thus showing a greater predisposition to skeletal class III. Interestingly, in non-operated adult cleft patients, Capelozza^30^ showed an increased ANB angle due to a protruded maxilla, thus suggesting that surgical treatment leads to restricted maxillary growth. In the BCLP and CPO groups, despite the presence of maxillary hypoplasia, the mandible was rotated posteriorly and diminished in size. As a result, the variables that affect the maxillomandibular relationship were not affected, thus contributing to camouflaging of the class III, which usually occurs in cleft palate patients.

In agreement with the results obtained by other researchers in operated^35^ and non-operated^30^ patients, the significantly higher gonial angle observed in the UCLP, BCLP, and CPO groups with respect to the controls in our study demonstrates a vertical pattern of the face in the cleft palate group. The vertical pattern is closely related to the characteristics of the palate. This is due to the low position of the tongue, which hinders the correct development of the maxilla, thus leading to a clockwise rotation of the mandible and resulting in a vertical facial growth pattern.

In concordance with the skeletal vertical growth pattern observed in the BCLP, UCLP, and CPO groups, frontal facial analysis showed an increase in the total vertical dimension and facial height in the UCLP and CPO groups and an increase in the facial height index in the BCLP group. The CL group did not show differences in any measure of the facial frontal analysis referring to vertical height, which corresponds to the group whose skeletal vertical growth pattern did not show changes with respect to the control.

The morphology of the cranial base in cleft palate patients is a matter of debate, with different results observed in the literature. A normal anterior cranial base is a common finding in many studies^14,36^, while a diminished anterior cranial base has been observed in others^30,32^. Actually, these differences may be attributable to discrepancies in the ethnicity and age criteria of the subjects. Our results in Spain show that there were no significant alterations of the cranial base in any of the groups. This validates the use in our study of cephalometric measurements that consider the cranial base, for example, SNA and SNB. A reduced cranial base could influence the development of maxillary hypoplasia due to the anatomical dependence of the bones that constitute the anterior cranial base and the maxilla. This applies equally to the saddle angle, which is another factor that could contribute to a more posterior position of the jaw. However, our results indicate that neither the cranial base nor this angle influenced the hypoplasia of the maxilla, which must be attributed to other factors. It also seems that neither the presence of the cleft nor the surgical treatment had any influence at the cranial base level.

Our study shows that, despite orthodontic treatment, the lower incisors were in linguoversion in the cleft groups except for the CL group. Lingual tipping of the lower incisors has been described in cleft palate patients, both unoperated^30^ and operated^35,37^. Regarding the upper incisors, the BCLP group was the only one that showed linguoversion. This inclination may be attributed to the altered function of the orbicularis oris muscle in cleft patients, in association with the presence of a scar due to upper lip repair, as reported previously^35,38^.

The internal canthi measurements reflect the position of the orbits at the skeletal level. Since Moss described an increase in the interorbital distance in unrepaired cleft patients^39^, different studies have focused their attention on hypertelorism in cleft patients, with inconsistent results. Hypertelorism seems to be more frequent in clefts affecting the primary palate^40,41^. However, the measurements performed on the facial photographs here did not show differences in the intercanthal width in the study group with respect to the control group.

Regarding the nasal soft-tissue analysis, we did not find any significant difference between the CL and the control groups. Using 3D photogrammetric analysis of the nose, Seo et al.^42^ found no differences between unilateral cleft lip patients and controls for the measurements analyzed, which is consistent with our results. Actually, 3D photogrammetric analysis might give more information than the 2D analysis used here, since it allows measurement of both area and volume^42^.

As specified by other authors^43,44^, in our study, the alar base width was significantly greater in the BCLP group than in the controls. This greater alar base width is characteristic of patients with BCLP and cannot be reduced with treatment^45^. Consequently, due to this greater nasal width, the ratio of columella height to alar base width was significantly lower in BCLP patients than in controls. Additionally, a greater alar base width was observed in the BCLP group than in CPO patients, and this led to a significantly lower ratio of columella height to alar base width in the BCLP group than in the CPO group. These results could also explain the significantly lower angulation of the left nostril angle in BCLP than in CPO patients, because with a wider nasal base, less angulation of the nostrils would be expected. Indeed, the fact that CPO patients did not show an alteration of the nose could mean that they behaved like controls for these purposes, thus justifying the significant differences between these two groups in these variables. However, it should be noted that significant differences were observed in the angulation of the nostrils (right versus left) in CPO patients. Lip impairment in patients of the CL group could condition some nasal alterations in this group and the non-appearance of significant differences of the nasal width between the BCLP and CL groups.

The UCLP patients also showed a statistically significant reduction of the ratio of columella height to alar base width with respect to the controls. However, in this case, the height of the columella and the width of the nasal base as independent variables, despite showing altered values, were not statistically significant, even when both characteristics appear typically in patients with UCLP^46^. In our study, this fact suggests that these measures alone were normalized with treatment, although the value of the ratio, which provides a relationship between the two measurements, failed to hide this trend.

Something similar occurred with the columella height to nasal height ratio, which was reduced both in the BCLP and UCLP groups with respect to the controls, although the measurements used to calculate this ratio (columella height and nasal height) alone were not significantly different in these groups. As stated by Wong et al.^47^, obtaining mathematical ratios reduces the error that results from any change in the distance between the photographer and the patient and provides an accurate result. Indeed, ratios are better than individual measurements because they avoid the effect of the subject’s height.

No significant differences were found among groups in the width of the mouth, thus indicating that a repaired CL does not affect the transversal development of the lips. However, in agreement with other researchers^28^, our results showed a significantly diminished upper lip length in the CL and BCLP groups compared to the control group, with the CL group showing the greatest decrease. In the BCLP group, this length was also significantly diminished with respect to the CPO group. This deficit is usually present before lip repair and may also be affected by lip surgery^28^.

In comparison with the results published by other authors, the lip and palate of the patients of our study were repaired later. The median and mean age values for cheiloplasty in our study were 5 and 10.2 months, respectively, and for palatoplasty they were 17 and 21.4 months, respectively. The most recommended age for cheiloplasty is between 3 and 6 months, whilst the age recommended for performing palatoplasty is between 6 and 18 months. We ignored the reason why the patients of our study had surgery later, since the Spanish guideline for cleft lip repair recommends that primary cheiloplasty be performed at the age of 3–6 months and palatoplasty, between 6 and 15 months^5^. This guideline, however, was published in 2011, when the patients of this study had already been operated on. On the other hand, we did not observe significant differences in the variables analyzed with regard to the age of the patients at the time of surgery. Differences in craniofacial growth between patients operated on early (before 4 to 5 years of age) or late (after 4–5 years) in childhood–adolescence have been found in other studies^48,49^, but all the patients in our study had primary surgery before or at 2 years of age.

The patients analyzed here underwent a total number of surgeries ranging between 1 and 11, with an average of 4.39. This number includes all types of fissures, although patients with BCLP had more surgeries than other groups, in consonance with the results obtained by other researchers^50,51^. Considering the burden for patients and their families, the figures reported here and in other studies seem to be high. The Sociedad Española de Fisuras Faciales (SOCEFF), Spanish association of facial cleft care providers, was created in 2000 aiming to improve protocols and teams to achieve good standards for cleft treatment and care. Most of the patients included in the present study were operated before the existence of this association or shortly after its inception. We believe that the gold standards for cleft treatment, as proposed by many researchers around the world^52^ are being taken into account, and, as in other countries, means to reduce the surgical burden of care are also in place in Spain, which will hopefully imply a decrease in the number of surgeries each cleft patient must undergo.

Since this study includes a retrospective analysis of surgical data, it has the limitations of gathering information that a prospective study would avoid. Therefore, the preoperative and operative data obtained from the medical records of the cleft patients are not complete, which precludes a conclusive analysis on the effect of the different pre-surgical and surgical techniques applied to the patients on the studied variables. Based on the limitations found in the present study, future prospective research should be performed in our country, taking special care to record pre-surgical and surgical data in the medical records of cleft patients. However, the analysis we have performed on the palate, craniofacial, and facial soft tissue of these patients at their maturity may serve as a control for future research in this field. Importantly, the fact that our results on craniofacial growth are largely consistent with those of other studies on the same type of cleft validates the use of an intraoral scanner to obtain measurements in the maxilla of cleft lip and palate patients. The characteristics and type of the cleft seem to be the major factors conditioning long-term craniofacial growth alterations in patients.

In conclusion, our results have shown alteration of the craniofacial pattern in the BCLP, UCLP, and CPO groups that essentially affect the palate, maxilla, and mandible, with a direct impact on facial growth and facial appearance. This condition was greater in the BCLP group than in the rest, reflecting the complexity of treatment and the consequences of this complex fissure. As might have been expected, the CL group presented a palate and craniofacial morphology very similar to that of the control group, with few measurements that differed between the two groups, essentially with respect to nasal measurements. Future prospective research must be planned in our country to clearly determine the effects of different types of treatment on the craniofacial appearance of cleft patients at their maturity.

## Methods

### Ethical statement

All procedures followed were in accordance with the ethical standards of the responsible committee on human experimentation (institutional and national) and with the Helsinki Declaration of 1964 and later versions. The study was approved by the Institutional Ethical Review Board of Hospital Clínico San Carlos of Madrid (project ID: 18/045-E_BC), and informed consent was obtained from all participants.

### Subjects

The sample of the study group consisted of 83 patients whose clefts were repaired early in childhood at the following Spanish hospitals: Hospital Clínico Universitario Virgen de la Arrixaca (Murcia), Hospital Universitario Miguel Servet (Zaragoza), Hospital Universitario Infantil Niño Jesús (Madrid), Complejo Hospitalario Universitario A Coruña (A Coruña), Complejo Hospitalario de Navarra (Pamplona), Hospital General Universitario Gregorio Marañón (Madrid), Hospital Universitario 12 de Octubre (Madrid), and Hospital Universitario de Burgos (Burgos).

For those patients for which we have data indicating when they had surgery with both cheiloplasty and palatoplasty (n = 37), 35 were operated on with cheiloplasty first, one was operated on with palatoplasty first, and one was operated with cheiloplasty and palatoplasty simultaneously.

The median age of the patients when cheiloplasty was performed (n = 47) was 5 months, with an average age of 10.2 months. The median age value for palatoplasty (n = 51) was 17 months, with a mean value of 21.4 months.

Different techniques were used for cheiloplasty, which mainly depended on the type of cleft and the preference of the surgeon. From the total of patients operated with any type of primary cheiloplasty (n = 57) in the participant hospitals, the technique most commonly used was Millard (n = 20, 35.1%), and, to a lesser extent, Malek (n = 3, 5.3%), Tennison-Randall (n = 2, 3.5%), Veau Axhausen (n = 2, 3.5%), and Manchester Mendoza (n = 1, 1.75%), with no data on the surgical technique used (n = 29) in 49.1% of the patients. Several patients required two or more cheiloplasties (n = 34). From those, the Mulliken and Fisher techniques were used in five (14.7%) and one (2.9%) patient, respectively. We have no data on the technique used in this case (n = 28) for 82.3% of the patients. From the total of patients operated on with any type of palatoplasty (n = 57) in the participant hospitals, the techniques used were the Sanvenero-Rosselli (n = 15, 26.3%), Veau Wardill Kilner "push-back" (n = 11, 19.3%), Von Langenbeck (n = 7, 12.3%), or Furlow technique (n = 1, 1.75%) or gingivoperiosteoplasty (n = 1, 1.75%), and we have no data on the technique used (n = 22) for 38.6% of the patients.

Of the patients from which we have data on nasal surgeries (n = 32), 18 (56.2%) were treated with primary rhinoplasty, whilst 14 (43.7%) needed secondary rhinoplasty or even more nasal surgeries. We have scarce information on the techniques used, but they were either open rhinoplasty, closed rhinoplasty or rhinoseptoplasty, using either osseous or cartilaginous grafts.

Patients underwent between 1 and 11 surgeries from infancy to maturity, with a median value of 4 and an average of 4.39.

Two surgeons per hospital operated on all the patients of each hospital. This sample was divided into four groups (Table 4): cleft lip (CL), unilateral cleft lip and palate (UCLP), bilateral cleft lip and palate (BCLP), and cleft palate only (CPO).

**Table 4:** Distribution of the sample according to sex and age.

	Control	CL	UCLP	BCLP	CPO
Total number of subjects (*n*)	71	6	37	16	24
Male/female (*n*)	28/43	1/5	20/17	6/10	12/12
Age (years, mean ± SD)	23.4 ± 4.3	23.6 ± 2.8	24.0 ± 3.6	22.9 ± 3.0	22.6 ± 2.8

*S.D.* Standard deviation.

The inclusion criteria for the study group were.

- Non-syndromic CL, UCLP, BCLP, or CPO at birth.
- Surgical repair of the cleft in a Spanish hospital collaborator in this study.
- No craniofacial congenital anomalies other than the cleft.
- No antecedents of craniofacial traumatism.
- No previous orthognathic surgery.
- Age between 18 and 30 years.
  The control group consisted of 71 subjects, all college students from Complutense University of Madrid (Table 4).

The inclusion criteria for the control group were

- Class I malocclusion.
- No crowding or minimal crowding of teeth.
- Previous lateral cephalogram
- No previous orthodontic treatment.
- No craniofacial congenital anomalies.
- No antecedents of craniofacial traumatism.
- Age between 18 and 30 years.

### Digital models

**Figure 1:**
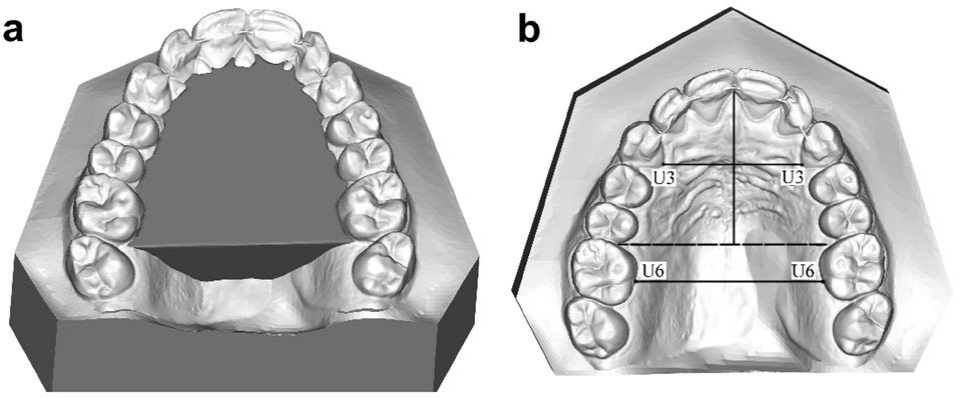
Analysis of 3D digital models. (**a**) Palatal volume. (**b**) Intercanine width (U3–U3) and intermolar width (U6–U6) measured at the gingival level. Arch depth is measured by tracing a line perpendicular to the dotted line joining the mesial aspect of the first molars. This line extends to the incisive papilla.

Digital models were obtained with a structured LED light intraoral scanner (CS 3600. Carestream, Rochester, NY, USA). STL files were imported to Nemocast (Nemotec, Madrid, Spain) for analysis. Figure 1 shows the 3D analysis of the models. The digital models were oriented in space using orthogonal lines, the medial raphe, and the occlusal functional plane. The software provides geometric information of the palatal volume. To calculate the palatal volume, a horizontal plane connecting the midpoints of the lingual gingival margin of the upper teeth was defined. The posterior limits of the palatal volume were the distal points of the upper first molars, where a plane perpendicular to the horizontal plane was established. The upper limit was the whole palatal surface. The volume was obtained by using Boolean operations. The intercanine width, intermolar width, and arch depth were also measured in the 3D models (Table 5). Intraoral scanning and 3D model measurements were performed by an orthodontist with 10 years’ experience using several intraoral scanners and 3D model analysis software.

**Table 5:** Landmarks for measurements on cephalometries and facial photographs.

Measurements	Landmarks
**Digital models**	
Maxillary intermolar width	U6–U6 (mm)
Maxillary intercanine width	U3–U3 (mm)
Arch depth	Line perpendicular to a line joining the mesial aspect of the first molars (mm). This line extends to the incisive papilla
**Maxilla**	
SNA	S-N-A (°)
Maxillary depth	Po-Or/N-A (°)
Midfacial length	Co-A (mm)
Point A to Nasion Perpendicular	A-N perp (mm)
**Mandible**	
SNB	S-N-B (°)
Facial depth angle	Po-Or/N-Pg (°)
Pogonion to Nasion perpendicular	Pg-N perp (mm)
Ramus height	Ar-Go (mm)
Mandibular body length	Go-Me (mm)
Effective mandibular length	Co-Gn (mm)
**Maxillomandibular relationship**	
ANB	A-N-B (°)
Maxillomandibular differential	Co-Gn minus Co-A (mm)
**Growth pattern**	
Facial axis	Pt-Gn/Ba-N (°)
Mandibular plane angle	Go-Gn/S-N (°)
Gonial angle	Ar-Go/Go-Me (°)
**Cranial base**	
Saddle angle	N-S-Ar (°)
Articular angle	S-Ar-Go (°)
Anterior cranial base	S-N (mm)
Posterior cranial base	S-Ar (mm)
**Incisor inclination**	
Lower incisor/mandibular plane (IMPA)	L1E-L1A/Go-Gn (°)
Upper incisor/Frankfort plane	U1E-U1A/Po-Or (°)
**Frontal soft-tissue analysis**	
Facial height	N′–Me′ (mm)
Facial width	Zy–Zy (mm)
Facial index	Facial height/Facial width × 100
Vertical dimension	Tr–Me′ (mm)
Width of the mouth	Ch_R-Ch_L (mm)
Intercanthal width	En_R-En_L (mm)
**Lateral soft-tissue analysis**	
Nasal length	N′-Sn (mm)
Nasolabial angle	C-Sn-Ls (°)
Upper lip position	Ls-E line (C-Prn) (mm)
Upper lip length	Sn-Stms (mm)
Lower lip length	Stmi-Me′(mm)
**Submental soft-tissue analysis**	
Alar base width	Al_R-Al_L (mm)
Nasal height	Sn-Prn (mm)
Columella height	Sn-Cs (mm)
Ratio of columella height to nasal height	Sn-Cs/Sn-Prn (ratio)
Ratio of columella height to alar base width	Sn-Cs/Al_R-Al_L (ratio)
Right nostril angle	Angulation of long axis of the right nostril (^°^)
Left nostril angle	Angulation of long axis of the left nostril (^°^)
Right nostril area	Area from the right nostril boundaries (mm^2^)
Left nostril area	Area from the left nostril boundaries (mm^2^)
Nasal deviation	Sn-Prn-facial midline (^°^)

### Skeletal and facial soft-tissue analysis

**Figure 2:**
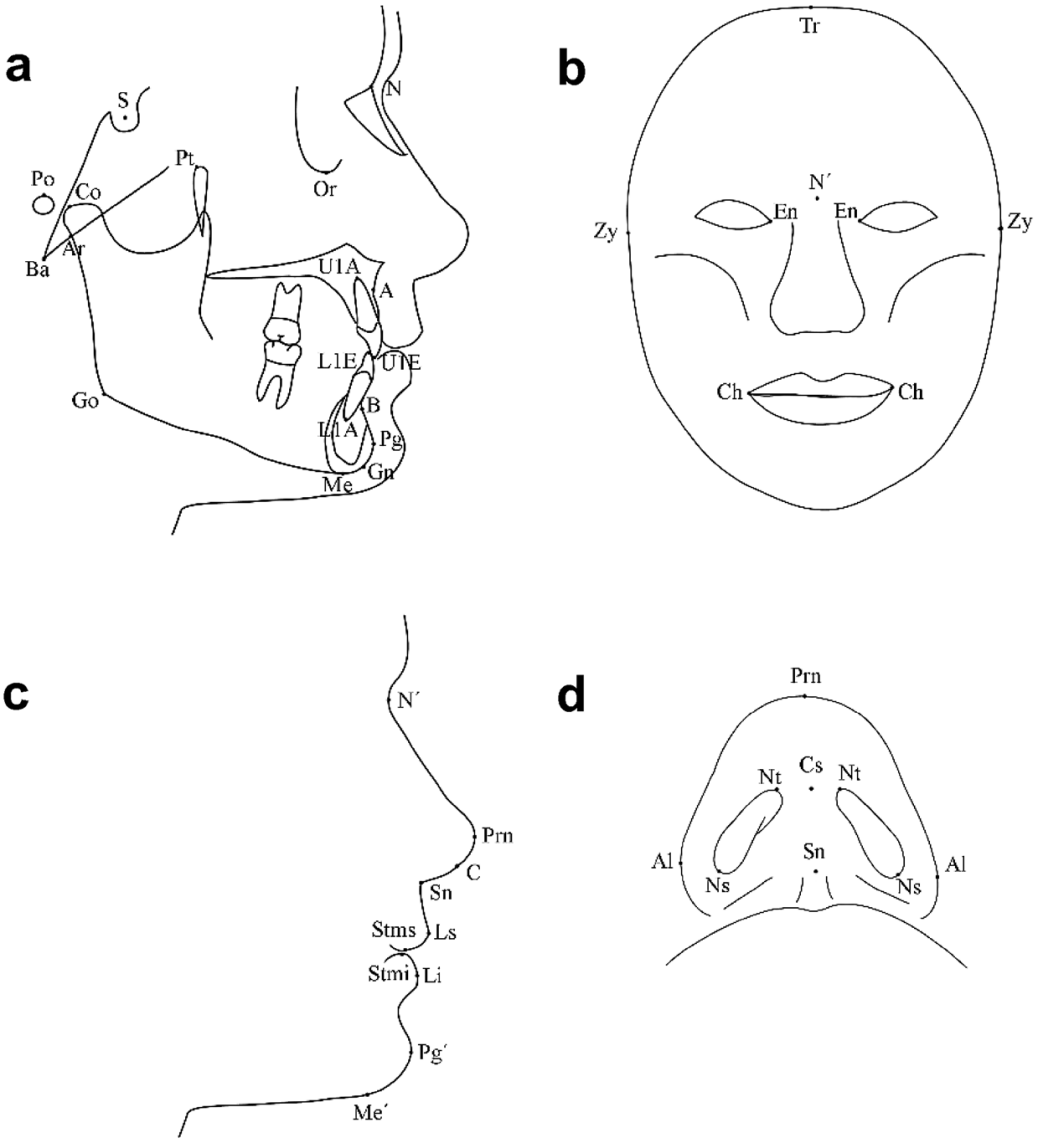
Landmarks on the lateral cephalogram (**a**) and frontal (**b**), lateral (**c**), and submental (**d**) facial photographs. *A* A point, *Al* alare, *Ar* articulare, *B* B point, *Ba* basion, *C* columella, *Cs* columella superius, *Ch* cheilion; *Co* condylion, *En* endocanthion, *Go* gonion, *Gn* gnathion, *L1A* lower central incisor apex, *L1E* lower central incisor edge, *Li* labrale inferius, *Ls* labrale superius, *Me* menton, *Me′* soft tissue menton, *N* nasion, *N′* soft tissue nasion, *Ns* nostril sill, *Nt* nostril tip, *Or* orbitale, *Pg* pogonion, *Pg′* soft tissue pogonion, *Prn* pronasale, *Po* porion, *Pt* pterygoid, *S* Sella, *Sn* subnasal, *Stmi* stomion inferior, *Stms* stomion superior, *Tr* trichion, *U1A* upper central incisor apex, *U1E* upper central incisor edge, *Zy* zygoma.

For this analysis, lateral radiographs and intraoral, frontal, lateral, and submental photographs were obtained. All radiographs and frontal and lateral photographs were taken in a natural head position with the teeth in centric occlusion. Landmark identification was performed on these records by an experienced investigator using a computerized tracing program (Nemoceph, Nemotec, Madrid, Spain). Figure 2 shows the landmarks recorded in the lateral cephalogram, the frontal facial photograph, the facial profile photograph, and the submental photograph of the nose. All records were calibrated before analysis. Table 5 shows the measurements and landmarks used for this part of the study.

Skeletal and dental variables were located on the lateral cephalogram. Variables were classified into the following groups for analysis and better comprehension: maxilla, mandible, maxillomandibular relationship, growth pattern, cranial base, and incisor inclination.

Analyses of the face, lips and eyes were performed on the frontal, lateral, and submental facial photographs. Lateral soft-tissue analysis of the lips and nose were performed on the lateral facial photograph. The submental view of the nose was obtained by orienting the head in such a manner that the tip of the nose was lined up between the internal canthi of the eyelids and the eyebrows. Image J software (NIH, Bethesda, MD, USA) was used to obtain measurements in this submental view.

### Statistical analysis

Statistical analysis was performed in the Computing Service and Research Support of Complutense University of Madrid using IBM SPSS 27 Statistics. The analysis of variables included the calculation of means and standard deviation for each variable in each group and the non-parametric Kruskal–Wallis test for comparison between groups. Symmetry of the nose, as well as right and left side nostril angles and areas were compared in all groups using the non-parametric Wilcoxon signed rank test. To analyze the influence of age of surgery on the variables, one-way analysis of covariance (ANCOVA) with Bonferroni-adjusted post hoc tests was used.

## Data Availability

Data supporting this study are available from the corresponding author (M.J.V.) upon reasonable request.
